# Drawing the line on in vitro gametogenesis

**DOI:** 10.1111/bioe.12679

**Published:** 2019-10-15

**Authors:** Lauren Notini, Christopher Gyngell, Julian Savulescu

**Affiliations:** ^1^ Melbourne Law School University of Melbourne Carlton Victoria Australia; ^2^ Biomedical Ethics Research Group Murdoch Children’s Research Institute Parkville Victoria Australia; ^3^ Department of Paediatrics University of Melbourne Carlton Victoria Australia; ^4^ The Oxford Uehiro Centre for Practical Ethics University of Oxford Oxford United Kingdom of Great Britain and Northern Ireland

**Keywords:** assisted reproductive technology, bioethics, in vitro‐derived gametes, in vitro gametogenesis, resource allocation, same‐sex reproduction

## Abstract

In vitro gametogenesis (IVG) might offer numerous research and clinical benefits. Some potential clinical applications of IVG, such as allowing opposite‐sex couples experiencing infertility to have genetically related children, have attracted support. Others, such as enabling same‐sex reproduction and solo reproduction, have attracted significantly more criticism. In this paper, we examine how different ethical principles might help us to draw lines and distinguish between ethically desirable and undesirable uses of IVG. We discuss the alleged distinction between therapeutic and non‐therapeutic uses of assisted reproduction in the context of IVG, and show how it is both problematic to apply in practice and theoretically dubious. We then discuss how the ethical principles of reproductive justice and beneficence apply to IVG for opposite‐sex reproduction, same‐sex reproduction, and solo reproduction. We suggest that these principles generate strong reasons for the use of IVG for opposite‐sex and same‐sex reproduction, but not for solo reproduction.

## 1 INTRODUCTION

In vitro gametogenesis (IVG1
IVG is a technology that creates gametes outside the body (in vitro) from somatic (non‐reproductive) cells, stem cells or progenitor cells: Hendriks, S., Dondorp, W., de Wert, G., Hamer, G., Repping, S., & Dancet, E. A. (2015). Potential consequences of clinical application of artificial gametes: A systematic review of stakeholder views. *Human Reproduction Update, 21*(3), 297–309. We use the more neutral terms ‘IVG’ and ‘in vitro‐derived (IVD) gametes’, rather than alternative terms used in the bioethics literature, such as ‘artificial’ or ‘synthetic’ gametes. This is because the terms ‘artificial’ or ‘synthetic’ are often value‐laden, owing to their association with the term ‘unnatural’, which may carry negative connotations: Douglas, T., Harding, C., Bourne, H., & Savulescu, J. (2012). Stem cell research and same‐sex reproduction. In M. Quigley, S. Chan & J. Harris (Eds.), *Stem cells: New frontiers in science and ethics* (pp. 207–228). Toh Tuck Link, Singapore: World Scientific; Smajdor, A., Cutas, D., & Takala, T. (2018). Artificial gametes, the unnatural and the artefactual. *Journal of Medical Ethics, 44*(6), 404–408. For similar reasons, we use the term ‘non‐IVD gametes’ instead of ‘natural gametes’.10.1093/humupd/dmv00225609402
) offers potential benefits, in both research and clinical contexts. In the research setting, IVG could improve current understanding of gametogenesis (gamete development) and various factors contributing to infertility.2
Ishii, T., & Saitou, M. (2017). Promoting in vitro gametogenesis research with a social understanding. *Trends in Molecular Medicine, 23*(11), 985–988; Segers, S., Mertes, H., de Wert, G., Dondorp, W., & Pennings, G. (2017). Balancing ethical pros and cons of stem cell derived gametes. *Annals of Biomedical Engineering, 45*(7), 1620–1632.10.1007/s10439-017-1793-928091967
 Knowledge gained from such research not only is valuable in and of itself, but also has the potential to lead to tangible clinical benefits, such as reduced infertility rates.3
Bredenoord, A. L., & Hyun, I. (2017). Ethics of stem cell‐derived gametes made in a dish: Fertility for everyone? *EMBO Molecular Medicine, 9*(4), 396–398.10.15252/emmm.201607291PMC537674428279974
 In vitro*‐*derived (IVD) gametes from various sources (including embryonic stem cells and induced pluripotent stem cells) have produced healthy and fertile offspring in non‐human animals, and human IVD gametes (both sperm and eggs) have been generated.4
Hendriks, S., Dancet, E. A., van Pelt, A. M., Hamer, G., & Repping, S. (2015). Artificial gametes: A systematic review of biological progress towards clinical application. *Human Reproduction Update, 21*(3), 285–296.10.1093/humupd/dmv00125609401
 IVG may one day be used by humans as an assisted reproductive technology (ART), changing the paradigm of genetic parenthood.

IVG is a new technological development, and its clinical applications in humans remain experimental.5
Ibid.
 It is unclear when, or if, IVG will be used in humans. One advantage of this is that there is additional time to analyse the novel ethical and social challenges that IVG poses.6
Bredenoord & Hyun, op. cit. note 3.
 As IVG becomes more of a technical possibility, serious consideration will need to be given to the ethical principles that should underlie decisions about which individuals should have access to this technology. Ethical consideration of IVG is also important to guide ongoing and future research.

Using IVG to allow opposite‐sex couples, in which one or both partners experience infertility, to have genetically related children has generally attracted support from bioethicists, members of the public and healthcare professionals.7
Hendriks et al., op. cit. note 1; Segers et al., op. cit. note 2; Hendriks, S., Dancet, E. A. F., Vliegenthart, R., & Repping, S. (2017). The acceptability of stem cell‐based fertility treatments for different indications. *Molecular Human Reproduction, 23*(12), 855–863.10.1093/molehr/gax02728460040
 Infertility may be due to various causes, including previous cancer treatment8
Ishii, T., & Pera, R. A. (2016). Creating human germ cells for unmet reproductive needs. *Nature Biotechnology, 34*(5), 470–473; Maltaris, T., Seufert, R., Fischl, F., Schaffrath, M., Pollow, K., Koelbl, H., & Dittrich, R. (2007). The effect of cancer treatment on female fertility and strategies for preserving fertility. *European Journal of Obstetrics and Gynecology and Reproductive Biology, 130*(2), 148–155.10.1016/j.ejogrb.2006.08.00616979280
 or medical conditions such as azoospermia (the inability to produce mature sperm9
Shabataev, V., & Tal, R. (2017). Artificial sperm: New horizons in procreation. *Rambam Maimonides Medical Journal, 8*(4), e0042; West, F. D., Shirazi, R., Mardanpour, P., Ozcan, S., Dinc, G., Hodges, D. H., … Nayernia, K. (2013). In vitro–derived gametes from stem cells. *Seminars in Reproductive Medicine, 31*(1), 33–38.10.1055/s-0032-133179523329634
) or premature ovarian insufficiency10
Chen, H. F., & Ho, H. N. (2018). Prospects of primary ovarian insufficiency patient‐specific pluripotent stem cells for disease modeling and clinical impacts. *Current Women's Health Reviews, 14*(1), 67–80.
 (where the ovaries cease to function completely or partially before the age of 40 years11
Batur, P., Budev, M. M., & Thacker, H. L. (2009). Women's hormonal health issues. In J. K. Stoller, F. A. Michoter Jr. & B. F. Mandell (Eds.), *The Cleveland Clinic intensive review of internal medicine* (5th ed., pp. 12–28). Philadelphia, PA: Wolters Kluwer Health/Lippincott Williams & Wilkins.
).

Other potential clinical applications of IVG, such as enabling same‐sex reproduction,12
Douglas et al., op. cit. note 1; Murphy, T. F. (2014). The meaning of synthetic gametes for gay and lesbian people and bioethics too. *Journal of Medical Ethics, 40*(11), 762–765; Segers, S., Mertes, H., Pennings, G., de Wert, G., & Dondorp, W. (2017). Using stem cell‐derived gametes for same‐sex reproduction: An alternative scenario. *Journal of Medical Ethics, 43*(10), 688–691.10.1136/medethics-2016-10386328122990
 postmenopausal motherhood,13
Cutas, D., & Smajdor, A. (2015). Postmenopausal motherhood reloaded: Advanced age and in vitro derived gametes. *Hypatia, 30*(2), 386–402.10.1111/hypa.12151PMC446107526074667
 ‘solo’ genetic parenthood or ‘solo IVG’14
‘Solo genetic parenthood’ or ‘solo IVG’ refers to the situation where an individual combines IVD gametes created from their somatic cells with their non‐IVD gametes to have a genetically related child, without involving a gamete donor: Cutas, D., & Smajdor, A. (2017). ‘I am your mother and your father!’ In vitro derived gametes and the ethics of solo reproduction. *Health Care Analysis, 25*(4), 354–369; Suter, S. M. (2016). In vitro gametogenesis: Just another way to have a baby? *Journal of Law and the Biosciences, 3*(1), 87–119. One potential group of individuals who might be interested in solo IVG are ‘single mothers by choice’, who may wish to eliminate the need for a gamete donor; Bock, J. D. (2000). Doing the right thing? Single mothers by choice and the struggle for legitimacy. *Gender & Society, 14*(1), 62–86. We thank anonymous Reviewer 1 for pointing this out.10.1007/s10728-016-0321-7PMC565670526969575
 and ‘multiplex parenting’,15
‘Multiplex parenting’ refers to the situation where IVD gametes are used to create a child who has more than two genetic parents. Palacios‐González, C., Harris, J., & Testa, G. (2014). Multiplex parenting: IVG and the generations to come. *Journal of Medical Ethics, 40*(11), 752–758.10.1136/medethics-2013-101810PMC421529124608087
 have attracted significantly more criticism.16
Hendriks et al., op. cit. note 1; Hendriks et al., op. cit. note 7; Segers et al., op. cit. note 2.



IVG could also be used to expand and accelerate genetic selection of offspring with favourable characteristics.17
Bourne, H., Douglas, T., & Savulescu, J. (2012). Procreative beneficence and in vitro gametogenesis. *Monash Bioethics Review, 30*(2), 29–48; Sparrow, R. (2014). In vitro eugenics. *Journal of Medical Ethics, 40*(11), 725–731; Shulman, C., & Bostrom, N. (2014). Embryo selection for cognitive enhancement: Curiosity or game‐changer? *Global Policy, 5*(1), 85–92.10.1007/BF03351338PMC359089923409535
 We will not consider this eugenic application in this paper, and instead focus on non‐eugenic ‘reproductive’ applications of IVG.

In this paper, we discuss the general ethical principles that might help to assess different clinical applications of IVG. We distinguish three broad clinical applications of IVG: opposite‐sex reproduction, same‐sex reproduction, and solo reproduction. For simplicity, we focus on the use of IVG for couples or individuals of normal reproductive age. However, we believe that most of what we write about the ethical principles governing these cases will also apply to other clinical applications, such as IVG for postmenopausal women.

We argue that, ultimately, decisions about whether individuals or couples should be permitted—and/or assisted— to access IVG depends on justice and welfare considerations; and that these considerations support the use of IVG by opposite‐sex and same‐sex couples. We begin by briefly describing how IVD gametes can be created.

## 2 TECHNIQUES FOR CREATING IVD GAMETES

Three main techniques for creating IVD gametes have been described in the literature. The primary difference among these techniques relates to the source of stem cells used to derive the IVD gametes. These sources include (1) induced pluripotent stem cells, (2) embryonic stem cells (ESCs) from cloned embryos, and (3) ESCs from in vitro fertilization (IVF) embryos.18
Segers et al., op. cit. note 2.
 Creating IVD gametes from induced pluripotent stem cells (iPSCs)19
Pluripotent stem cells are non‐embryonic stem cells that can become any body cell, excluding embryonic and placental tissue: Halim, A. S., & Mohaini, M. (2013). Adipose‐derived stem cells in tissue engineering: Laboratory to bedside. *JUMMEC: Journal of Health and Translational Medicine, 16*(2), 1–10.
 involves genetically reprogramming a somatic cell (e.g. skin cell) from one of the prospective parents to become iPSCs, and differentiating these iPSCs into IVD gametes, which can be combined with other IVD gametes (or non‐IVD gametes) in vitro. IVD gametes can also be created from ESCs taken from cloned embryos20
The cloned embryo is the genetic clone of the prospective parent who provided the somatic cell. This embryo is used to create the IVD gametes; it is not the embryo that becomes the resulting child.
 created using somatic cell nuclear transfer.21
Bourne et al., op. cit. note 17.
 Finally, IVD gametes can be derived from ESCs taken from embryos created through IVF.22
This initial IVF embryo would be created by combining a non‐IVD gamete from one of the prospective parents with a non‐IVD gamete from a gamete donor. This embryo (embryo 1) will be destroyed once ESCs have been taken from it and differentiated into IVD gametes.
 This approach avoids some of the technical challenges23
Several technical obstacles need to be overcome to generate functional male gametes from females in humans (and vice versa). This is primarily due to the phenomenon known as ‘imprinting’. For sperm to function correctly, they need special epigenetic markers (that turn genes on or off) that are inherited from the paternal line. Similarly, for oocytes to function correctly, they need epigenetic markers inherited from the maternal line. Sperm created from female somatic cells will lack these ‘imprinted’ marks, and likewise for oocytes derived from male somatic cells. This would also make the use of IVG for solo reproduction close to impossible, because that requires male and female gametes from one individual.
 associated with the other two methods.24
Segers et al., op. cit. note 12.
 The IVD gamete from one prospective parent would be combined in vitro with a non‐IVD gamete from the other prospective parent, and the resulting child would share one‐half of their DNA with the parent who provided the non‐IVD gamete, one‐quarter with the parent who provided the IVD gamete, and one‐quarter with the gamete donor.

## 3 THREE CLINICAL APPLICATIONS OF IVG

### 3.1 IVG for opposite‐sex reproduction

Infertility is commonly defined as a failure to become pregnant after one year of trying to conceive via unprotected sexual intercourse.25
World Health Organization. (2018). *Infertility definitions and terminology.* Retrieved from http://www.who.int/reproductivehealth/topics/infertility/definitions/en/ [Accessed Jun 28, 2016].
 Approximately one in six opposite‐sex couples meets this definition of infertility. There are many causes of infertility, including genetic factors, infections, and social factors (such as delaying conception).

In many countries, opposite‐sex couples experiencing infertility have access to a range of ARTs. Some ARTs, such as assisted insemination, have existed for centuries. Others, such as IVF, are more recent. IVF is now the most commonly used form of ART.26
Padubidri, V. G., & Daftary, S. N. (2015). Infertility and sterility. In V. G. Padubidri & S. N. Daftary (Eds.), *Shaw's textbook of gynaecology* (16th ed., pp. 237‐262). New Delhi, India: Elsevier.
 In some countries, current figures estimate that 6% of all children have been conceived via an ART, primarily IVF.27
Nyboe Andersen, A., & Erb, K. (2006). Register data on assisted reproductive technology (ART) in Europe including a detailed description of ART in Denmark. *International Journal of Andrology, 29*(1), 12–16.10.1111/j.1365-2605.2005.00577.x16466519
 While IVF is effective for many couples, there are still many gaps; approximately 50% of opposite‐sex couples who access IVF remain childless after treatment.28
van Loendersloot, L., Repping, S., Bossuyt, P. M. M., van der Veen, F., & van Wely, M. (2014). Prediction models in in vitro fertilization; Where are we? A mini review. *Journal of Advanced Research, 5*(3), 295–301; Malizia, B. A., Hacker, M. R., & Penzias, A. S. (2009). Cumulative live‐birth rates after in vitro fertilization. *New England Journal of Medicine, 360*(3), 236–243.10.1056/NEJMoa080307219144939



Some people lack the internal capacity to produce any gametes, or lack so few functioning gametes that establishing a pregnancy is nearly impossible. IVG will provide a novel treatment for such cases.

### 3.2 IVG for same‐sex reproduction

Compared with alternative family‐building options currently available to same‐sex couples, IVG is unique as it could enable *both* partners to be the genetic parents of the resulting child. This benefit would apply to both same‐sex male29
We use the term ‘male’ to refer to a person who has XY chromosomes, whether or not they have functioning testes or sperm. Similarly, we use the term ‘female’ to refer to an individual who has XX chromosomes, regardless of whether they have functioning ovaries or eggs. We acknowledge that some individuals may not identify with their ‘chromosomal sex’. For example, a person with XX chromosomes may identify as male, and a person with XY chromosomes may identify as female. They may also identify as intersex or non‐binary. The terms ‘male’ and ‘female’ are used in this paper for the sake of simplicity.
 and female couples.30
Same‐sex female couples may also have the option to reproduce using mitochondrial replacement: Baylis, F. (2013). The ethics of creating children with three genetic parents*. Reproductive Biomedicine Online, 26*(6), 531–534. However, mitochondrial replacement offers far less of a genetic relation to the resulting child for one of the mothers compared with IVG, as mitochondrial DNA constitutes less than 0.1% of the total DNA. For this reason, it seems reasonable to speculate that most same‐sex female couples wanting to reproduce genetically with each other would opt for IVG over mitochondrial replacement, although such a hypothesis has, to our knowledge, not been tested empirically.10.1016/j.rbmo.2013.03.00623608245
 Intra‐familial gamete donation would also enable both individuals in a same‐sex couple to be genetically related to the resulting child (e.g. the biological sister of one of the male partners could donate her eggs); hence, IVG is not unique in this regard. However, if IVG using somatic cells is used, both intended parents (the parent who provides the somatic cell and the parent who contributes the non‐IVD gamete) would contribute approximately 50% of their DNA to the child and would therefore both be considered the genetic parents.31
Segers et al., op. cit. note 2.
 If IVG using ESCs from IVF embryos is used, one parent would contribute 50% of their DNA to the child and the other would contribute 25%, as described earlier.

### 3.3 IVG for solo reproduction

The most novel potential clinical application of IVG is that it enables solo reproduction. This is theoretically possible, if both male and female gametes are produced from a single individual. These gametes could be combined *in vitro*, using IVF technologies. Females with a functional uterus could carry the child themselves, while others could use a surrogate.

Solo reproduction is a common form of reproduction in nature. In nature, solo reproduction relies on the process of mitosis, where a single cell produces genetically identical copies of itself (clones). IVG will make possible a novel form of solo reproduction, which relies on the process of meiosis rather than mitosis. Meiosis involves a shuffling of the genetic material in each cell, producing genetically distinct gametes. If these gametes are combined with each other, the result will be genetically distinct products, rather than clones.

## 4 ETHICAL PRINCIPLES FOR IVG

These different potential clinical applications of IVG raise the normative question of how each application should be ethically assessed. Are some clinical applications more ethically desirable than others? Despite various papers describing ethical issues raised by IVG,32
Bredenoord & Hyun, op. cit. note 3; Segers et al., op. cit. note 2; Smajdor, A., & Cutas, D. (2015). *Nuffield Council on Bioethics: Background paper: Artificial gametes* Retrieved from http://nuffieldbioethics.org/wp-content/uploads/Background-paper-2016-Artificial-gametes.pdf [Accessed Mar 8, 2018]; Mertes, H., & Pennings, G. (2010). Ethical aspects of the use of stem cell derived gametes for reproduction. *Health Care Analysis, 18*(3), 267–278; Testa, G., & Harris, J. (2005). Ethics and synthetic gametes. *Bioethics, 19*(2), 146–166.10.1007/s10728-009-0136-x19813094
 much normative work needs to be done in analysing the potential implications of the various clinical uses of this technology and identifying ethical principles that might help us to prioritize different clinical applications.

### 4.1 The alleged distinction between therapeutic and non‐therapeutic uses of ART

One explanation for why people may be comfortable with the idea of using IVG for opposite‐sex reproduction33
Hendriks et al., op. cit. note 1; Hendriks et al., op. cit. note 7.
 is that this is seen as a therapeutic intervention provided for medical reasons and an extension of current ARTs. In contrast, requests for ARTs for solo or same‐sex reproduction are often considered to be non‐therapeutic in nature (i.e. motivated by social, rather than medical, reasons) and therefore ethically suspect.34
This argument is mentioned, although not endorsed, by Segers et al., op. cit. note 2.



This idea that a distinction between therapeutic and non‐therapeutic interventions can distinguish between acceptable and unacceptable uses of IVG relies on two central claims:

Claim 1: Using IVG to enable opposite‐sex reproduction within normal reproductive age would be therapeutic in nature (i.e. provided for medical reasons), whereas using IVG to enable same‐sex or solo reproduction would be non‐therapeutic in nature (i.e. provided for social reasons).

Claim 2: There are stronger moral reasons to allow or promote interventions provided for medical, rather than social, reasons.

However, both claims are highly problematic, as we explain below.

### 4.2 Therapeutic versus non‐therapeutic IVG

The idea that using ARTs for same‐sex reproduction is non‐therapeutic is common.35
Cavaliere, G., & Palacios‐González, C. (2018). Lesbian motherhood and mitochondrial replacement techniques: Reproductive freedom and genetic kinship. *Journal of Medical Ethics, 44*(12), 835–842; Baylis, F. (2018). 'No' to lesbian motherhood using human nuclear genome transfer. *Journal of Medical Ethics, 44*(12), 865–867.10.1136/medethics-2017-104450PMC628869729491042
 For example, in the context of mitochondrial donation, Francoise Baylis has claimed: ‘While the initial goal of mitochondrial replacement technology is therapeutic insofar as it aims to avoid the birth of a child with mitochondrial disease, this technology could be used without a therapeutic intent. For example, it could be used to pursue non‐therapeutic reproductive goals—imagine, a lesbian couple where both partners wanted a genetic link to the children they intend to parent’.36
Baylis, op. cit. note 30, p. 533.



Therapeutic interventions, or interventions provided for medical reasons, are deemed to align with the proper goals of medicine. Conversely, non‐therapeutic interventions, or interventions provided for social reasons, are not. The question of whether a particular intervention is therapeutic or non‐therapeutic in nature therefore turns on what the proper goals of medicine are. The general consensus is that the proper goal of medicine is to serve patient health.37
Oakley, J., & Cocking, D. (2001). A virtue ethics approach to professional roles. In J. Oakley & D. Cocking (Eds.), *Virtue ethics and professional roles* (pp. 74–94). Cambridge, U.K.: Cambridge University Press.
 Hence, whether we should consider an application of a technology therapeutic or not ultimately depends on which theory of health and disease we endorse.

According to one prominent theory of health known as ‘the biostatical theory’, a disease is defined as something that causes deviation from normal functioning, with ‘normal functioning’ understood as a statistically typical contribution to survival and reproduction.38
Boorse, C. (1977). Health as a theoretical concept. *Philosophy of Science, 44*(4), 542–573; Boorse, C. (2014). A second rebuttal on health. *Journal of Medicine and Philosophy, 39*(6), 683–724.10.1093/jmp/jhu03525398760
 It is easy to see how the use of IVG for opposite‐sex couples experiencing infertility can be deemed therapeutic on this view, as its aim is to provide39
Foer example in the case of azoospermia.
 or restore40
For example in the case of medical infertility due to cancer treatment.
 fertility, an aspect of ‘normal’ functioning.

Conversely it could be argued that enabling same‐sex or solo reproduction does not restore normal functioning, and constitutes an intervention provided for social, rather than medical, reasons. This argument may then be used to justify allowing opposite‐sex couples, but not same‐sex couples and individuals who want to achieve sole genetic parenthood, access to IVG. Medical providers are only obliged to provide interventions for medical, not social, reasons, in keeping with the proper goals of medicine.41
Segers et al., op. cit. note 2.



However, as highlighted elsewhere, when circumstances are considered, the situation of some infertile opposite‐sex couples is not much different from the situation of same‐sex couples. For example, consider the following case.

*(Kelly & Zack):* Kelly and Zack, an opposite‐sex couple, attend a fertility clinic for a consultation. They wish for a child who is genetically related to both of them, but are unable to achieve this without clinical assistance. Kelly has an extremely low number of eggs, and Zack produces very few sperm. Both Kelly and Zack could have a genetic child without assistance if they reproduced with a different partner who does not have any fertility issues. However, it is near‐impossible for Kelly and Zack to conceive without assistance as a couple. Kelly and Zack knew of each other’s fertility issues before they became a couple.42
This case has been adapted from Douglas et al., op. cit. note 1, p. 213.

‘Kelly’ and ‘Zack’ can be regarded as ‘situationally infertile’,43
Murphy, T. F. (2018). Pathways to genetic parenthood for same‐sex couples. *Journal of Medical Ethics, 44*(12), 823–824. Published Online First: 27 April 2017. 10.1136/medethics-2017-104291.28450403
 as they are both capable of reproducing without assistance with any other individual, just not each other.

Note, though, the similarity between Kelly and Zack and a same‐sex couple, ‘Jill and Jessica’. Both Jill and Jessica have the physical ability to produce gametes and reproduce with other people. As a couple, however, they have no chance of conceiving a child together. They are situationally infertile in the same way as Kelly and Zack.

This reflects the fact that we often consider infertility a property of *couples* rather than of individuals, such as in the clinical definition of infertility endorsed by the World Health Organization44
World Health Organization, op. cit. note 25, unpaginated, emphasis added.
, namely: ‘Infertility is the inability of a sexually active, non‐contracepting *couple* to achieve pregnancy in one year’. Infertility in couples can result not just from the functioning of the individuals’ bodies, but also from the situation they find themselves in. Just as cases of ‘situational’ infertility are considered sufficient grounds for opposite‐sex couples to access ARTs (potentially including IVG in the future), so too should it be for same‐sex couples.

The use of IVG for solo reproduction seems like a much clearer case of an intervention requested for social, rather than medical, reasons. Still, even in this case an argument can be made that facilitating solo reproduction via IVG is therapeutic. Consider an asexual person, Bill. Bill produces functional gametes and has the physical ability to have children with others. While Bill has no sexual attraction to others, he desires genetically related children. This is a relatively common position for many asexual individuals, who are happy to rear children with others in whom they may be romantically, but not sexually, interested. Bill is distinct in that he has a strong desire to not share this genetic relationship with his future children with another person—that is, he has a strong desire to pursue solo reproduction.

It is clear that Bill’s asexuality is in some sense an abnormal function under Boorse’s account of health.45
Boorse, op. cit. note 38.
 It is not contributing to his survival or reproduction. The use of IVG to help Bill have genetically related children could be seen as restoring a natural function (reproduction) and therefore could be provided for medical, rather than social, reasons.

Hence it is not at all clear that, under a ‘normal functioning’ view of health, the use of IVG (and other ARTs) for opposite‐sex reproduction is any more therapeutic than it is for same‐sex or solo reproduction once we admit other models of relationships, including solo parent–child relationships.

If we move away from a ‘normal functioning’ account of health, there are further reasons to believe that uses of IVG beyond opposite‐sex reproduction can legitimately be considered therapeutic in nature. For example, take a broad normative account of health, such as the one given by the World Health Organization, which defines health as ‘a state of complete physical, mental, and social well‐being and *not merely the absence of disease or infirmity*’.46
World Health Organization. (2006). Constitution of the World Health Organization. Retrieved from http://www.who.int/governance/eb/who_constitution_en.pdf, p. 1 (emphasis added) [Accessed Jun 28, 2018].
 Under this broad definition of health, IVG can be deemed an intervention provided for medical reasons if it promotes psychosocial wellbeing, regardless of the person or couple using it.

Infertility, whether physical or situational, may give rise to harmful psychological and social consequences.47
Douglas et al., op. cit. note 1; Thompson, K., & McDougall, R. (2015). Restricting access to ART on the basis of criminal record. *Journal of Bioethical Inquiry, 12*(3), 511–520.10.1007/s11673-015-9622-z25701147
 For example, some studies have shown that the incidence of mental health conditions such as anxiety and depression is higher among women in opposite‐sex couples seeking ART than in women in the general population.48
Chen, T. H., Chang, S. P., Tsai, C. F., & Juang, K. D. (2004). Prevalence of depressive and anxiety disorders in an assisted reproductive technique clinic. *Human Reproduction, 19*(10), 2313–2318; Wright, J., Duchesne, C., Sabourin, S., Bissonnette, F., Benoit, J., & Girard, Y. (1991). Psychosocial distress and infertility: Men and women respond differently. *Fertility and Sterility, 55*(1), 100–108.1986949
 Opposite‐sex couples in which one or both partners experience infertility also report feeling stigmatized, lacking control over their lives, psychological distress, low self‐esteem, anger, shame and insecurity.49
Cousineau, T. (2007). Psychological impact of infertility. *Best Practice & Research Clinical Obstetrics & Gynaecology, 21*(2), 293–308; Valentine, D. P. (1986). Psychological impact of infertility: Identifying issues and needs. *Social Work in Health Care, 11*(4), 61–69; Pasch, L. A., & Sullivan, K. T. (2017). Stress and coping in couples facing infertility. *Current Opinion in Psychology, 13*, 131–135; Monga, M., Alexandrescu, B., Katz, S. E., Stein, M., & Ganiats, T. (2004). Impact of infertility on quality of life, marital adjustment, and sexual function. *Urology, 63*(1), 126–130.10.1016/j.urology.2003.09.01514751363
 There is less research on the effects of infertility on same‐sex couples. However, it may be that same‐sex couples experience less distress in relation to their infertility compared with opposite‐sex couples. This is because the biological limitations that prevent same‐sex couples from reproducing together without the assistance of technology would presumably prevent same‐sex couples from having the same expectations about having genetically related children together as opposite‐sex couples. In other words, if same‐sex couples have fewer expectations regarding having genetically related children with their chosen partner, same‐sex couples may experience much less infertility‐related distress than is experienced by opposite‐sex couples, who may feel that they need to live up to social expectations to have genetically related children with their partners.50
We thank anonymous Reviewer 2 for raising this point.
 Nevertheless, as some same‐sex couples desire shared genetic parenthood, it stands to reason that the ability to have genetically related children will also greatly benefit same‐sex couples, even if they may experience less infertility‐related distress than opposite‐sex couples.

We know of no empirical work that details anxiety and stress experienced by people who desire solo genetic reproduction. However, as individuals are even less likely to have expectations about genetically reproducing *with themselves*, any distress that is associated with not being able to reproduce asexually may not be particularly great.51
We thank anonymous Reviewer 2 for raising this point.
 Hence, while IVG may also promote the wellbeing of those who wish to be solo genetic parents, it may do so to a lesser degree than in the case of opposite‐sex and same‐sex couples. If those who wish to be solo genetic parents do indeed experience less infertility‐related distress owing to their having fewer expectations about reproducing, the health‐related reasons for allowing this group to access IVG are less strong than those for making IVG available to opposite‐sex and same‐sex couples.

It is also important to note that the dividing line between physical functioning and psychosocial wellbeing may not always be a sharp one. For example, it is possible that an improvement in the patient’s psychosocial wellbeing by assisting them to have a genetically related child may translate to indirect improvements in that patient’s physical functioning. An individual who has a genetically related child through ART may experience less stress52
We leave open the possibility that the individual may in fact experience more stress; namely, the potential stressors associated with raising a child.
 and therefore experience better physical health.

In sum, it is difficult to distinguish between different applications of IVG by employing the (alleged) distinction between interventions provided for medical and social reasons. More importantly, it is doubtful that such a distinction bears any moral significance.

### 4.3 Moral difference between interventions provided for medical and for social reasons

Even if we could usefully segregate potential clinical applications involving IVG into those motivated by medical versus social reasons, it is doubtful that this alone could help to prioritize these different applications. The question of whether morally relevant differences exist between interventions requested for medical and for social reasons has been frequently debated in the bioethics literature.53
Savulescu, J. (2007). Genetic interventions and the ethics of enhancement of human beings. In B. Steinbock (Ed.), *The Oxford handbook of bioethics* (pp. 516–535). New York, NY: Oxford University Press; Gyngell, C., & Selgelid, M. J. (2016). Human enhancement: Conceptual clarity and moral significance. In S. Clarke, J. Savulescu, C. A. J. Coady, A. Giubilini & S. Sanyal (Eds.), *The ethics of human enhancement: Understanding the debate* (pp. 111–126). New York, NY: Oxford University Press.
 We will therefore not go into the details of these debates here, but simply note that it is prima facie unhelpful to simply appeal to the view that ‘therapeutic interventions (motivated by medical reasons) should be provided and non‐therapeutic interventions (motivated by social reasons) should not’ without further argument. In our view, there are few ways in which this distinction can be usefully employed to help us draw lines and decide between ethically desirable and undesirable uses of IVG.

### 4.4 Reproductive justice

Some authors have appealed to justice as an important ethical principle when assessing the different potential clinical applications of ARTs.54
Murphy, op. cit. note 43; Harris, J. (2007). Reproductive choice. In John Wiley & Sons, Ltd (Ed.), *Encyclopedia of life sciences* Chichester, UK: John Wiley. 10.1002/9780470015902.a0005595




At its core, justice requires giving ‘each their due’.55
Cicero, M. T. (1933). *On the nature of the gods. Academics *Translated by H. Rackham. Loeb Classical Library 268. Cambridge, MA: Harvard University Press.
 For different levels of access to the same resource to be considered fair, these must be based on morally relevant differences between patient groups. In other words, it is fair to give different people different levels of access to the same resource, if morally relevant differences exist between them. While not equal, this would be considered equitable and therefore fair. For access to a resource to be considered fair and equitable, all patients in similar circumstances should be granted similar levels of access.

Hence, the question then becomes, do any morally relevant differences exist between individuals wishing to use IVG for same‐sex, opposite‐sex or solo reproduction, such that it is fair to grant the former group access to IVG, but not the latter groups? If so, differing levels of access can be considered fair and equitable. If not, different levels of access can be considered unjust discrimination.

It is clear that opposite‐sex individuals experiencing infertility have a claim on access to IVG. Owing to features of their biology, they have been denied a good that others are free to enjoy (having genetically related children). Because many people value a biological relationship with their offspring, this is an injustice. These same considerations also apply to same‐sex couples and to individuals who desire solo reproduction. Owing to differences in their sexual orientation (which are either legitimate choices or beyond their control) or single status, they are not able to have genetically related children with their preferred partner (or, in the case of solo reproduction, themselves).

It is sometimes implied that there is an inherent worth in what is ‘natural’,56
President's Council on Bioethics. (2002). *Human cloning and human dignity: An ethical inquiry*. Washington, DC: National Bioethics Advisory Commission.
 and that this can ground a right to activities such as opposite‐sex reproduction, but not other forms of reproduction. But such appeals have been shown to be highly problematic in this context.57
Buchanan, A. (2011). Human nature and the natural. In A. E. Buchanan (Ed.), *Beyond humanity?: The ethics of biomedical enhancement* (pp. 115–142). Oxford, U.K.: Oxford University Press.
 The concept of human ‘nature’ is notoriously difficult to pin down, and may be particularly difficult to apply to IVG.58
Smajdor et al., op. cit. note 1.
 Moreover, it can be argued that those with genetic causes of infertility should not ‘naturally’ reproduce—IVF is the most unnatural of interventions itself, even for opposite‐sex couples.

Looking at *historical* injustice may give us reasons to give preference to some groups over others. This point is strongly made by Timothy Murphy: ‘It’s plausible in some ways that same‐sex59
This appears as ‘opposite‐sex’ couples in Murphy’s original publication. It has been confirmed through personal communication with the author that this was intended to be ‘same‐sex couples’.
 couples are owed research priority towards securing shared genetics in their children simply as a matter of access and equity and also—more searchingly—as a matter of compensatory justice, for past road blocks imposed against having children.’60
Murphy, op. cit. note 43.



Same‐sex couples have traditionally been persecuted in many societies, and have had few opportunities for family building. This may place a general duty on society to support their attempts to have genetically related children. Because same‐sex couples have been wronged in such societies, society might have reasons of justice to remedy these past wrongs, including by enabling them to build families through IVG.

One could argue that, because single‐parent families have also been wrongfully shamed and penalized throughout history and single individuals are not permitted to access ART in many jurisdictions,61
See, for example, Riezzo, I., Neri, M., Bello, S., Pomara, C., & Turillazzi, E. (2016). Italian law on medically assisted reproduction: Do women’s autonomy and health matter? *BMC Women’s Health, 16*(1), 44.10.1186/s12905-016-0324-4PMC495841027449932
 it seems that single individuals have an equal justice‐based claim to ART as opposite‐sex and same‐sex couples.62
We thank anonymous Reviewer 1 for raising this point.
 While we agree that single individuals also have a justice‐based claim to ART, we disagree that this claim is sufficiently strong to warrant permitting such individuals to access solo IVG.

To our knowledge, very few individuals have been identified who have reproductive desires involving solo genetic reproduction. Furthermore, single individuals can attain genetic parenthood using existing ARTs or via unassisted reproduction. If single individuals are excluded from accessing existing ARTs in some jurisdictions, it seems likely that these same individuals would also be excluded from accessing IVG. Genetic parenthood cannot be attained without IVG for both partners in a same‐sex couple, or for both partners in an opposite‐sex couple who lack the ability to produce functioning gametes. Prohibiting IVG for single individuals who wish to reproduce asexually would merely mean that these individuals cannot be the *only* genetic parent. Prohibiting IVG for opposite‐sex couples in which both partners lack functioning gametes or for same‐sex couples would mean that both partners in these couples would never be the genetic parent of the same child. This morally relevant difference—in terms of the options that being denied access to IVG would close off for the individuals involved—means that opposite‐sex and same‐sex couples have a greater claim to IVG than those who wish to be solo genetic parents.

In sum, a justice‐based argument can be made that same‐sex couples should have access to or even be prioritized in the development and distribution of IVG. Conversely, as those who wish to be solo genetic parents can still attain genetic parenthood (albeit not *solo* genetic parenthood) via other means, a justice‐based argument for IVG is not as applicable for solo genetic reproduction.

### 4.5 Beneficence and non‐maleficence

#### 4.5.1 Benefits to users

As detailed above, involuntary infertility can take a huge toll on an individual’s or couple’s psychosocial wellbeing. Numerous studies have found that infertility is related to depression, anxiety, feelings of isolation, lowered self‐esteem, and stress.63
Cousineau, op. cit. note 49; Greil, A. L., Slauson‐Blevins, K., & McQuillan, J. (2010). The experience of infertility: A review of recent literature. *Sociology of Health & Illness, 32*(1), 140–162.10.1111/j.1467-9566.2009.01213.xPMC338379420003036
 One study of 200 infertile opposite‐sex couples found that 15% of the men and half of the women stated that infertility was the ‘most upsetting experience of their lives’.64
Freeman, E. W., Boxer, A. S., Rickels, K., Tureck, R., & Mastroianni, L., Jr. (1985). Psychological evaluation and support in a program of in vitro fertilization and embryo transfer. *Fertility and Sterility, 43*(1), 48–53, p. 48.10.1016/s0015-0282(16)48316-03965315
 For many, the most difficult aspect of infertility is a loss of agency—a loss of control over one’s life.65
Domar, A. D., & Seibel, M. M. (1997). Emotional aspects of infertility. In M. M. Seibel (Ed.), *Infertility: A comprehensive text* (pp. 29–44). Stamford, CT: Appleton & Lange.
 IVG will provide a way for people to take back this control. We have strong reasons of beneficence to allow the use of IVG for opposite‐sex couples experiencing infertility.

As we have argued earlier, same‐sex couples who wish to share genetic parenthood would also likely experience great benefits from IVG. Hence, there are also reasons of beneficence to allow the use of IVG for same‐sex couples. However, if same‐sex couples do indeed experience less infertility‐related distress than opposite‐sex couples, owing to having fewer expectations regarding having genetically related children with their chosen partner, these beneficence‐based reasons may be less strong in the case of same‐sex couples.

Some may argue that same‐sex couples have other family‐building options (such as adoption and gamete donation), and access to IVG will therefore yield them little additional benefit. However, the same argument can be made in relation to opposite‐sex couples who pursue IVF currently; they could pursue adoption or gamete donation instead. Furthermore, IVG is distinctive in that it is the only ART that could allow same‐sex couples to have an equal genetic relationship with their child. This would also be true for opposite‐sex couples in which one or both partners cannot produce gametes (for example, as a result of chemotherapy‐induced infertility), and therefore for whom IVF is not a viable option. Hence, IVG may also be the only ART that would allow some opposite‐sex couples to genetically reproduce together. In this respect, same‐sex reproduction via IVG may not be too different from these examples of opposite‐sex reproduction.66
We thank anonymous Reviewer 2 for raising this point.



In addition to the psychosocial harms associated with (physical/medical or situational) infertility, additional psychosocial harms may arise from the unequal genetic relationships with one’s child that existing ARTs result in for same‐sex couples, or for opposite‐sex couples who pursue gamete donation. For example, jealousy regarding non‐equal genetic parenthood has been reported by some same‐sex female couples.67
Pelka, S. (2009). Sharing motherhood: Maternal jealousy among lesbian co‐mothers. *Journal of Homosexuality, 56*(2), 195–217.10.1080/0091836080262316419197649



One might counter that in such cases adoption is a preferable option—neither partner is genetically related to their child. However, obstacles remain to pursuing adoption, particularly for same‐sex couples in some jurisdictions. Moreover, while the value of genetic relatedness is difficult to cash out in objective terms,68
Savulescu, J. (2003). The public interest in embryos. In J. Gunning, & H. Szoke (Eds.). *The regulation of assisted reproductive technology legislation* (pp. 191–202). Aldershot, U.K.: Ashgate.
 it remains true that many people value it highly and that it forms a dominant part of many autonomous life plans.

If IVG becomes an ART option, this could increase any infertility‐related distress that same‐sex couples experience, strengthening beneficence‐based reasons for permitting same‐sex couples to access IVG. Social norms about family building have arguably already changed for same‐sex couples, and could be expected to change even further if IVG becomes available. These changes in social norms could raise the expectations of individuals in same‐sex couples about having children with their chosen partner, and therefore increase the distress they experience in relation to their situational infertility. For example, as same‐sex marriage has become more socially accepted, and as more same‐sex couples use ARTs to build their families (even if both parents are not genetically related to the child, as is the case with currently available ARTs for same‐sex couples), it is likely that individuals in same‐sex couples have increased their expectations about building a family with their partner. If IVG does become an available ART option, this could increase the hopes and expectations of same‐sex‐attracted individuals about having genetically related children with their partner. This could ultimately mean that same‐sex couples experience the same kind and level of distress in relation to their infertility as that currently experienced by many infertile opposite‐sex couples.69
We thank anonymous Reviewer 2 for raising this point.



As we argued earlier, infertility‐related distress would be unlikely to be particularly great in the case of individuals desiring solo genetic parenthood, given that individuals are not likely to have expectations about genetically reproducing with themselves. Hence, beneficence‐based reasons for facilitating access to IVG seem least strong in the case of solo IVG. Nonetheless, it remains possible that there are some people, like Bill described above, for whom solo genetic reproduction is important to their life plans, and who would benefit from the provision of IVG in this way.

#### 4.5.2 Concerns about harm to children

One concern about IVG relates to the future child’s wellbeing. IVG carries the risk of introducing harmful genetic mutations.70
Ishii & Pera, op. cit. note 8; Master, Z. (2006). Embryonic stem‐cell gametes: The new frontier in human reproduction. *Human Reproduction, 21*(4), 857–863; Moreno, I., Míguez‐Forjan, J. M., & Simón, C. (2015). Artificial gametes from stem cells. *Clinical and Experimental Reproductive Medicine, 42*(2), 33–44; Mouka, A., Tachdjian, G., Dupont, J., Drévillon, L., & Tosca, L. (2016). In vitro gamete differentiation from pluripotent stem cells as a promising therapy for infertility. *Stem Cells and Development, 25*(7), 509–521.10.1089/scd.2015.023026873432
 This is less of a risk with the use of intermediate embryos and ESCs, as we described. There are safety risks involved with any technology, particularly new technologies. At present, we cannot accurately estimate the level of risk that IVG would present. One way of addressing this concern is to ensure that IVG is sufficiently safe before permitting its clinical use. This would require rigorous safety trials, monitoring and follow‐up, beginning with studies with non‐human animals (which have already commenced). Another way to address concerns about the safety of IVG is to permit IVD gametes to be created from IVF‐ESCs but not from somatic cells, as the latter approach requires more cellular manipulations and therefore more risk.71
Segers et al., op. cit. note 2.



That said, some uses of IVG are inherently riskier than others. The most obvious risky application of IVG is its use in solo reproduction. Solo reproduction via IVG will be an extreme case of inbreeding—the equivalent of identical twins conceiving with each other. IVG for solo reproduction will be different from cloning, as the assortment of alleles at meiosis increases the chance that deleterious heterozygous mutations are brought to homozygosity in the offspring.72
Testa & Harris, op. cit. note 32.
 It has been estimated that each human carries one or two genetic mutations. If two copies of the same genetic mutation are inherited, this can lead to severe genetic disease or prenatal death.73
Genetics Society of America (2015, Apr 8). Hidden burden: Most people carry recessive disease mutations. *Science News* Retrieved from https://www.sciencedaily.com/releases/2015/04/150408100522.htm [Accessed May 31, 2019].
 If the same individual were producing gametes (non‐IVD or IVD gametes) for solo genetic reproduction, there would be a 25% chance that the resulting child would inherit two copies of these one or two genetic mutations (i.e., they would be homozygous for these genetic mutations). This is because there would be a 50% chance that the solo genetic reproducer’s egg would have the genetic mutation, and a 50% chance that their sperm would have the same mutation.74
This situation would be like two carriers of a genetic mutation reproducing with one another, which also carries a 25% risk that the resulting child will be homozygous for that mutation.
 These significant health risks to the resulting child generate strong reasons against the use of IVG for solo reproduction.

In our view, these significant health risks to the resulting child constitute the most ethically salient objection against IVG for solo reproduction. This distinguishes our ethical position on solo IVG from that of other authors, such as Suter,75
Suter, op. cit. note 14.
 who has argued that, while solo IVG is *potentially* ethically problematic, whether or not solo IVG is *actually* ethically problematic in a specific case is dependent on the individual’s underlying motivations for pursuing solo IVG. Suter's argument suggests that there are ethically problematic and ethically understandable motivations for wanting to pursue solo IVG.

In the hypothetical case of Bill we described earlier, we noted that Bill wishes to pursue solo IVG as he has a strong desire to not share a genetic relationship with his future child with another person. Bill’s underlying motivations for not wanting to share genetic parenthood are not stated, but would be worth exploring with Bill as they may carry some ethical significance.

For example, imagine that Bill reveals that his parents went through a messy divorce when he was a child and the family endured a lengthy, drawn‐out custody battle. Bill is determined to avoid the possibility that his future child might go through a similar experience, and he states that the only way to completely prevent this is if he is the only genetic parent of his child. If this were indeed Bill’s underlying motivation, psychosocial support should be offered to Bill to help him work through these issues from his childhood, which may be unresolved. That is, Bill’s motivation is ethically significant from a beneficence point of view; it highlights clinicians’ obligations to offer additional support to Bill.

In addition, Bill’s motivation is ethically significant for reasons relating to informed consent. Information would need to be provided to Bill about the legal rights of gamete donors in his particular jurisdiction. It may be that a contract can be drawn up ahead of time to ensure that an egg donor would not be able to claim custody rights. Bill would also need to be informed that, even if he does pursue solo IVG, a third party (the gestational carrier) would still be involved. In some jurisdictions, courts have granted gestational carriers parental rights, despite the fact that they are not the genetic or intended mother of the child.76
For example, *In re* T.J.S., 54 A.3d 263 (N.J. 2012).
 Hence, solo IVG may not completely alleviate Bill’s concerns about fighting over custody of the resulting child.

Bill’s underlying motivation for wanting to exclude others from having a genetic relationship with his child is ethically salient, in the sense that addressing his concerns and providing tailored information may result in Bill deciding not to pursue solo IVG any further. However, Bill’s underlying motivation for pursuing solo IVG is *not* the most ethically salient aspect of this case. The most ethically salient concern is the significant health risks to the resulting child, which we have described earlier. These risks are so likely, and of such great magnitude, that *no* underlying motivation for pursuing solo IVG can justify them, no matter how laudable the motivation itself. While some motivations for pursuing solo IVG may make certain cases more ethically problematic than others, all instances of solo IVG are sufficiently ethically problematic (owing to the health risks to the child) that they should not be permitted according to our ethical analysis. In other words, the ethically problematic nature of solo IVG does not depend on an individual’s motivations for wanting to pursue it, but rather on the significant health risks that solo IVG poses for the resulting child.

Appeals to harms to children conceived via ARTs run into well‐known concerns about the ‘non‐identity problem’. Say Bill uses IVG for solo reproduction, and this results in a child with a moderate disability—Ben. If Bill did not use IVG, Ben would not exist. It therefore seems that Ben has not been made worse off by Bill’s decision to use IVG; hence, we cannot appeal to Ben’s interests to judge Bill’s use of IVG impermissible.

However, just because Ben is not made worse off in a counterfactual sense by Bill’s action to pursue solo reproduction does not mean that this action is permissible. Parfit famously drew attention to harmless wrongdoing or impersonal harm.77
Parfit, D. (1984). *Reasons and persons.* Oxford, U.K.: Clarendon Press.
 One of us (JS) has argued that while it may be (impersonally) wrong to deliberately select a deaf embryo, it may nonetheless be permissible because it does not harm the offspring in person‐affecting terms in virtue of the non‐identity problem.78
Savulescu, J. (2002). Deaf lesbians, "designer disability," and the future of medicine. *British Medical Journal, 325*(7367), 771–773.10.1136/bmj.325.7367.771PMC112427912364309
 However, where there is a significant risk of producing an offspring with disabilities so profound that they render life not worth living, then person‐affecting and impersonal considerations would speak against allowing such reproduction. Inbreeding, particularly within one individual, would have a significant chance of producing profound genetic abnormalities. For this reason, arguably, we ought not to allow solo reproduction using IVG.

Furthermore, when assessing the potential applications of IVG from a policy perspective, it is even clearer that such harms are relevant to the assessment. When comparing different public health policies, for example, if one policy was likely to increase the number of people experiencing a moderate disability, this fact is relevant to assessment of that policy, even if the people with the disability would not have existed if the policy was not in place.

The fact that solo reproduction through IVG is an extreme form of inbreeding generates strong moral reasons against pursuing this application of IVG. From this point, we will consider IVG for same‐sex, but not for solo, reproduction.

Some authors have expressed concern that a child raised by same‐sex parents is worse off than a child raised by opposite‐sex parents.79
Anderson, R. T. (2013, Mar 11). Marriage: What it is, why it matters, and the consequences of redefining it. The Heritage Foundation Backgrounder, 2775. Retrieved from https://www.heritage.org/marriage-and-family/report/marriage-what-it-why-it-matters-and-the-consequences-redefining-it [Accessed Jun 28 2018]; Somerville, M. A. (2003, April 29). The case against 'same‐sex marriage'. A brief submitted to the Standing Committee on Justice and Human Rights. Retrieved from http://eng1020detroit.pbworks.com/w/file/fetch/51612757/CaseAgainstChangingDefinition.pdf [Accessed Jun 30, 2018]
 This concern may be due to a belief that opposite‐sex couples make better parents than same‐sex couples, because fathers allegedly have parenting skills that mothers do not, and vice versa.80
This argument is mentioned, but not endorsed, by Douglas et al., op. cit. note 1.
 In this case, the harms focus is not on adverse physical health consequences, but on the negative social consequences that would purportedly stem from having two parents of a single gender. For example, Margaret Somerville argues that same‐sex reproduction violates a sexual ecology that is important to the welfare of children, and undermines a social symbolism essential in the transmission of life.81
Somerville, op. cit. note 79.
 This concern is not unique to IVG, and has been articulated in other contexts where same‐sex couples wish to raise children, such as in the case of adoption.82
Somerville, op. cit. note 79; Hayes, B. C. (1997). the influence of gender on public attitudes toward homosexual rights in Britain. *International Journal of Public Opinion Research, 9*(4), 361–385.



Whether children of same‐sex couples are worse off than comparable children of opposite‐sex couples is ultimately an empirical question. The evidence seems to show very clearly that they are not.83
Knight, K. W., Sarah, E. M., West, S., Martin, B., Jones, C. A., Little, M. H., … Wake, M. (2017). The kids are OK: It is discrimination, not same‐sex parents, that harms children. *Medical Journal of Australia, 207*(9), 374–375.10.5694/mja17.0094329092695
 A recent meta‐analysis of 40 studies of children raised by same‐sex couples concluded that these children fared just as well as other children across multiple wellbeing measures, including academic performance, cognitive development, social development, and psychological health.84
Manning, W. D., Fettro, M. N., & Lamidi, E. (2014). Child well‐being in same‐sex parent families: Review of research prepared for American Sociological Association Amicus Brief. *Population Research and Policy Review, 33*(4), 485–502.10.1007/s11113-014-9329-6PMC409199425018575
 It seems reasonable to expect that the same would hold true for children born to same‐sex couples via IVG.

Therefore, while considerations of the interests of children provide reasons against solo reproduction using IVG, there is no case for this to count against same‐sex couples using the same technology.

#### 4.5.3 Harm to society

Potential harms may also arise for society in general. Permitting the use of IVG may perpetuate the problematic idea that genetic parenthood is superior to other types of parenthood. This could translate to physically or situationally infertile individuals continuing to experience the psychosocial harms associated with non‐genetic parenthood. Paradoxically, in seeking to alleviate infertility‐related distress, IVG could actually increase infertility‐related distress for those who are unable to access it.85
We thank anonymous Reviewer 2 for raising this point.
 For example, if many same‐sex couples opt for IVG, this could mean that same‐sex couples who do not or who are unable to access this technology (e.g. owing to lack of financial resources) experience even more psychosocial harm than they would have, if the new expectation is that same‐sex couples will avail themselves of this technology to attain shared genetic parenthood. Hence, IVG seemingly raises a dilemma in that it seeks to address a problem (infertility‐related distress) that it could also perpetuate.86
We thank anonymous Reviewer 2 for raising this point.
 This dilemma, however, seems to be one of resources, rather than of IVG itself. If IVG were to become available as an ART option, fertility clinics would need to be prepared for the likely onslaught of requests they would receive. If resources are an issue and not all who wish for IVG can receive it,87
It seems likely that resources would be less of an issue for IVG than for some existing ARTs; for example, if IVD gametes were created using somatic cells, this would address the current donor gamete (particularly egg) shortage that exists in many countries.
 fertility clinics would need to have just allocation systems in place. IVG could also be publicly funded, as some existing ARTs are in some jurisdictions. A detailed discussion of what such allocation and funding systems could look like in the context of IVG is beyond the scope of this paper.

We agree with the claim that society’s emphasis on genetic parenthood is a primary factor contributing to individual preferences for genetic children and the psychosocial harms associated with infertility. Society should recognize that genetic parenthood is not required to be a ‘good parent’, and that how parents raise their children is just as important as any genetic relationship, if not even more so.88
Zeiler, K., & Malmquist, A. (2014). Lesbian shared biological motherhood: The ethics of IVF with reception of oocytes from partner. *Medicine, Health Care and Philosophy, 17*(3), 347–355.10.1007/s11019-013-9538-524395218
 However, it cannot be denied that many individuals who are infertile experience psychosocial distress related to (even if not directly caused by) their infertility. To change the existing social preference for genetic parenthood is no small task and would take many years, if not decades or centuries, to achieve. As Murphy states, genetic parenthood is still regarded (rightly or wrongly) as ‘a cultural gold standard’.89
Murphy, op. cit. note 43.
 In the interim, many people will continue to suffer from the psychosocial harms associated with infertility. IVG can help to alleviate these psychosocial harms. Furthermore, even if such social change occurs, it is reasonable to expect that many individuals will still prefer to have genetic children.

Another potential societal harm of clinical IVG is that it may devalue families who are socially, rather than genetically, connected. Allowing IVG could be socially harmful if it diminishes the efforts of socially connected, but not genetically connected, families to have their families regarded as equally valid and deserving of respect as genetically connected families.90
We thank anonymous Reviewer 2 for raising this point.
 This includes families in which the child is not genetically related to either social parent, as well as families in which the child is genetically related to one social parent.91
Suter, op. cit. note 14.
 Some of these families may have deliberately prioritized social connectedness over genetic relatedness in their creation; consider, for example, an opposite‐sex couple who can genetically reproduce but who chose not to because they wanted to adopt instead. Other socially connected, but not genetically connected, families may have preferred to be genetically connected but could not pursue this option (for example, if both partners lacked functioning gametes, or if the couple could not access ARTs).

It is important to note that the concern that offering IVG could privilege genetically connected families over other types of families is not unique to IVG but can also be levelled against existing forms of ARTs, such as IVF, intrauterine insemination and intracytoplasmic sperm injection. This same concern could even be leveraged against allowing individuals to reproduce genetically without assistance. Despite raising similar concerns about perpetuating the ‘ideal’ of genetic parenthood and despite the existence of alternative family‐building options (e.g. adoption, donor gametes), these ARTs are routinely facilitated (and, in some jurisdictions, publicly funded), and non‐assisted reproduction is not generally regarded as problematic. This is because the wish for a child who is genetically related to both partners is typically seen as a reasonable goal for opposite‐sex couples. For reasons of consistency, the same should also hold for same‐sex couples.92
Segers et al., op. cit. note 2; Douglas et al., op. cit. note 1; Zeiler & Malmquist, op. cit. note 88.



IVG itself would not devalue socially connected, but not genetically connected, families. Rather, if such families were regarded as inferior, this would represent a broader social problem. There are many different types of families, and socially connected, but not genetically connected, families are just as important as genetically connected families. Many couples value having a genetic connection with their child, including couples who wish to genetically collaborate with their partner, and are curious as to which of their genetic traits will be passed on to their child. This is evident from those who go through costly and sometimes painful ARTs.93
Suter, op. cit. note 14.
 Other couples do not pursue this genetic connection, and prefer or are satisfied with social connections alone. The type of family that an individual or couple prefers is a personal choice, and does not necessarily mean that they do not value other types of families. Offering IVG as a clinical option would suggest that the choice to have a genetically connected family is a legitimate choice, just as is the choice to have a socially connected, but not genetically connected, family. To promote choice in family building, it is not sufficient to merely offer IVG. Rather, existing unacceptable obstacles to achieving parenthood through non‐genetic means (such as adoption) also need to be removed. In addition, laws must protect families who are socially, but not genetically, connected.94
Ibid.
 Promoting choice in family‐building is best served by breaking down existing barriers for those who wish to pursue socially connected, but not genetically connected, families, rather than by creating new barriers for those who wish to build genetically connected families.

Arguably, making IVG available as an ART option does nothing to address existing social problems, including those relating to children who need a family but are not being adopted. Some may therefore question whether IVG is the best use of resources.95
We thank anonymous Reviewer 2 for raising this point.
 While we are sympathetic to such concerns, it is important to again note that these concerns are not unique to IVG but also apply to existing ARTs. Furthermore, it is likely that even if there were no ARTs, many couples would still wish to reproduce genetically and would therefore not consider adoption. In this respect, unassisted reproduction also does nothing to address existing social problems relating to children who are in need of, but do not have, a home.96
Indeed, to some degree it is arguably contributing to these problems.
 Hence, the large numbers of children who are not being adopted is a broader social issue; while IVG may perpetuate this social issue to some degree, so do existing family‐building options, including unassisted reproduction. Again, this highlights the importance of continuing with broader social efforts to address these issues (including more education about various family‐building options—including those that do not involve genetic relatedness—and breaking down unacceptable barriers to adoption and gamete or embryo donation). These efforts may take place alongside offering IVG and other ARTs for those who wish to build their families via these methods.

The potential future widespread clinical availability of IVG will also challenge traditional concepts of the family. For example, one question that has been posed in the bioethics literature is whether a man who provides an IVD egg can really be thought of as the child’s father.97
Newson, A. J., & Smajdor, A. C. (2005). Artificial gametes: New paths to parenthood? *Journal of Medical Ethics, 31*(3), 184–186.10.1136/jme.2003.004986PMC173410115738444
 If the relationship between the resulting child and each of their parents is unclear, this could lead to psychosocial distress in the child. However, this concern remains speculative and could presumably be mitigated depending on when and how the process is explained to the child involved.

It is also important to note that this possibility is not unique to IVG and has already arisen in other contexts. For example, the American Thomas Beatie, the world’s first ‘pregnant man’ whose story attracted worldwide media attention, is a transgender man whose eggs allowed him to achieve both social fatherhood and genetic parenthood with his children.98
Murphy, op. cit. note 12; Beatie, T. (2008). *Labor of love: The story of one man’s extraordinary pregnancy.* Berkeley, CA: Seal Press.
 Hence, there are already cases where a genetic and gestational ‘mother’ is actually the child’s father, and traditional conceptions of the family may no longer be tenable or appropriate. The Nuffield Council on Bioethics has recommended that one potential way of addressing these concerns

…is for legislators to let go of the expectation that each child has two parents, one mother (preferably who contributed eggs) and one father (preferably who contributed sperm), and generally to revise the legal framework based on different criteria….[s]hould legislators choose to focus on family functioning instead, then, intricacies such as…male genetic mothers…would lose a lot of their weight.99
Smajdor & Cutas, op. cit. note 32, p. 14.

## 5 CONCLUSION

In this paper, we have shown the various reasons why the alleged distinction between therapeutic and non‐therapeutic interventions is not useful in distinguishing between ethically desirable and undesirable uses of IVG. Depending on which theory of health one endorses, it is possible to make the case that IVG is therapeutic for opposite‐sex couples, same‐sex couples and those who wish to pursue solo reproduction. We have shown that using the ethical principles of justice and beneficence is more fruitful in allocation decisions. We have argued that there are strong reasons of compensatory justice to allow same‐sex couples to access IVG, more so than opposite‐sex couples and those who wish to pursue solo reproduction. There are also beneficence‐based reasons to allow opposite‐sex couples, same‐sex couples and those who wish to pursue solo reproduction to access IVG, as this will alleviate the psychosocial harms associated with infertility and non‐equal genetic parenthood. However, we have claimed that arguments that IVG may help alleviate distress and improve wellbeing may not be as applicable for individuals who wish to be solo genetic parents, as these individuals presumably have fewer expectations regarding reproduction compared with opposite‐sex and same‐sex couples. Furthermore, there are countervailing harm‐based reasons to not allow IVG for solo reproduction, owing to the significant risks to the resulting child. So long as these risks exist, IVG should not be permitted for solo reproduction. Moving forward, priority should be given to researching IVG for opposite‐sex and same‐sex reproduction. Furthermore, if and when IVG becomes available, opposite‐sex and same‐sex couples should be prioritized in access.

## CONFLICT OF INTEREST

The authors declare no conflict of interest.

